# Staging of prostate Cancer with ultra-fast PSMA-PET scans enhanced by AI

**DOI:** 10.1007/s00259-024-07060-7

**Published:** 2025-01-11

**Authors:** David Kersting, Katarzyna Borys, Alina Küper, Moon Kim, Johannes Haubold, Tsepo Goerttler, Lale Umutlu, Pedro Fragoso Costa, Jens Kleesiek, Christoph Rischpler, Felix Nensa, Ken Herrmann, Wolfgang P. Fendler, Manuel Weber, René Hosch, Robert Seifert

**Affiliations:** 1 Department of Nuclear Medicine and German Cancer Consortium (DKTK), University Hospital Essen, University of Duisburg-Essen, Hufelandstraße 55, Essen, 45147 Germany; 2 Institute for Artificial Intelligence in Medicine, University Hospital Essen, Essen, Germany; 3 Institute of Interventional and Diagnostic Radiology and Neuroradiology, University Hospital Essen, Essen, Germany; 4 Cancer Research Center Cologne Essen (CCCE), University Medicine Essen, Essen, Germany; 5 German Cancer Consortium (DKTK), Partner Site Essen, Essen, Germany; 6 Department of Nuclear Medicine, Klinikum Stuttgart, Stuttgart, Germany; 7 Department of Nuclear Medicine, Inselspital, Bern University Hospital, University of Bern, Bern, Switzerland

**Keywords:** Digital PET, PET/CT, Artificial Intelligence, Low-count PET, Image post-reconstruction

## Abstract

**Purpose:**

PSMA-PET is a reference standard examination for patients with prostate cancer, but even using recently introduced digital PET detectors image acquisition with standard field-of-view scanners is still in the range of 20 min. This may cause limited access to examination slots because of the growing demand for PSMA-PET. Ultra-fast PSMA-PET may enhance throughput but comes at the cost of poor image quality. The aim of this manuscript is to evaluate the accuracy of AI-enhanced ultra-fast PSMA-PET for staging of patients with prostate cancer.

**Methods:**

A total number of 357 whole-body [^68^Ga]Ga-PSMA-11 PET datasets were included. Patients underwent two digital PET scans, one at standard and one at ultra-fast speed (table speed: 0.6–1.2 mm/s vs. 50 mm/s). A modified pix2pixHD generative adversarial network to enhance the ultra-fast images was trained with 286 datasets and evaluated with the remaining 71 datasets. The staging accuracy of ultra-fast PSMA-PET and AI-enhanced ultra-fast PET was compared with the reference standard PET separately for miTNM regions proposed by PROMISE V2.0.

**Results:**

The AI-network significantly improved the visual image quality and detection rate in most miTNM regions compared with the non-enhanced image data (T: 69.6% vs. 43.5%, *p* < 0.05; N: 46.3% vs. 27.8%, *p* < 0.01; M1a 64.4% vs. 47.5%, *p* < 0.01; M1b: 85.7% vs. 72.1%, *p* < 0.01). However, improvement was not significant for the M1c category (42.9 vs. 28.6%, *p* > 0.05). Missed lesions had a smaller SUVmax and lesion size compared with detected lesions (exemplary for N: 9.5 vs. 26.5 SUVmax; 4 vs. 10 mm). SUVmax values of lesions were significantly different in all miTNM regions between the ultra-fast and reference standard PET, but only in the T-region between the AI-enhanced and reference standard PET.

**Conclusion:**

The AI-based image enhancement improved image quality and region detection rates by a mean of 17.9%. As the sensitivity of synthetic PET for small and low-uptake lesions was limited, a potential clinical use case could be disease monitoring in patients with high tumor volume and PSMA uptake undergoing PSMA radioligand therapy. The improvement in detection rate of distant metastases was not significant. This indicates that more training data is needed to ensure robust results also for lesions that have lower appearance frequency. Future studies on accelerated PSMA-PET seem warranted.

**Supplementary Information:**

The online version contains supplementary material available at 10.1007/s00259-024-07060-7.

## Introduction

Staging and restaging of patients with prostate cancer is routinely done with prostate-specific membrane antigen (PSMA) targeted PET imaging. Prospective trials have shown its superior performance for the primary staging of high-risk prostate cancer, biochemical recurrence, or local lymph node staging in intermediate and high-risk prostate cancer [1–3]. Moreover, PSMA-PET is used in advanced stages of castration resistant prostate cancer to assess PSMA expression as a prerequisite for eligibility of radioligand therapy or to monitor the disease burden and decide on treatment intensification [4–7]. Recently, the PROMISE framework has been introduced, which facilitates a standardized reporting of PSMA-PET [8]. It comprises a comprehensive analysis of PSMA expression and location and, therefore, enables easy communication with referring physicians. To this end, the miTNM staging system was integrated into PROMISE, which is oriented on the pathological TNM system of the AJCC and reports suspicious PSMA accumulations separately for local tumor, local lymph nodes and distant disease [8, 9].

The FDA approval of PSMA ligands has increased the demand for this imaging modality [1, 2, 10, 11]. However, the scanning time of a standard field-of-view PET scanner is approximately 20 min. This potentially limits the access to PSMA-PET and could therefore delay treatment optimization. Also, repeated PSMA-PET examinations are performed for restaging purposes, which raises the need to minimize the radiation exposure of patients.

To address these needs, digital PET systems have been introduced, which offer higher sensitivity and better image quality compared with conventional PET systems [12–17]. In addition, machine learning techniques have been proposed to enhance the image quality of accelerated PET scans [18–21]. For example, we could show in a prospective trial that the acceleration of FDG-PET by a factor of approximately 30 was feasible and that an AI-network could improve the image quality by denoising the ultra-fast PET images [22]. However, such ultra-fast PET acquisitions have so far not been adopted for PSMA-PET. Also, the accuracy of such AI-assisted image improvement has not been assessed according to the miTNM framework, which allows for a structured and quantitative assessment of clinically relevant parameters.

The aim of the present work was, therefore, to assess the feasibility and accuracy of ultra-fast PSMA-PET scanning and image enhancement by a dedicated AI-network. To this end, the miTNM-compliant staging of patients with prostate cancer was performed with both ultra-fast and AI-enhanced ultra-fast (synthetic) PET images and compared with standard PSMA-PET acquisitions (as reference). Moreover, the influences of lesion size and tracer uptake were evaluated.

## Materials and methods

### Patients

All patients with prostate cancer who were scheduled for a clinical [^68^Ga]Ga-PSMA-11 PET/CT scan on a digital Biograph Vision 600 PET/CT system (Siemens Healthineers, Erlangen, Germany) at our department (Department of Nuclear Medicine, University Hospital Essen, Essen, Germany) between January 2020 and June 2020 were offered enrollment in this prospective, single-arm, single-center phase I/II imaging study. Inclusion criterion was age ≥ 18 years. The study was approved by the local ethics committee (Ethics committee, University Duisburg-Essen, Faculty of Medicine, protocol number 20-9226-BO) and performed in accordance with the Declaration of Helsinki. Written informed consent was requested. 357 male patients who underwent [^68^Ga]Ga-PSMA-11 PET/CT were enrolled. Mean ± standard deviation (SD) patient age was 70 ± 8 years and mean ± SD weight was 88 ± 16 kg.

### PET/CT image acquisition and reconstruction

PET/CT data were acquired 56 ± 16 min (mean ± SD) after administration of 108 ± 19 MBq (mean ± SD) of [^68^Ga]Ga-PSMA-11. Clinical PET/CT acquisition and reconstruction was performed according to our established routine PET protocol for [^68^Ga]-based PET tracers on this digital PET system [14]. In brief, first a CT scan in full-dose or low-dose technique (depending on the clinical availability of prior full-dose CT images) was acquired. PET data (at standard acquisition time) were acquired in continuous bed motion mode with a table speed velocity of 1.2 mm/s; the prostate region was emphasized by a reduced table speed velocity of 0.6 mm/s. The resulting mean ± SD whole-body PET acquisition time was 13.5 ± 1.6 min. For this study, between CT and standard PET acquisition a separate ultra-fast PET data set was acquired at a table speed velocity of 50 mm/s - this is the fastest possible option on the Biograph Vision 600 PET/CT system (with a 26.3-cm field-of-view). For all PET image reconstructions, a three-dimensional Poisson ordered-subsets expectation maximization algorithm with time-of-flight option was applied. 4 iterations and 5 subsets, a 2-mm Gaussian filter, and a 220 matrix (corresponding voxel size 3.3 × 3.3 × 3.0 mm^3^) were used. All PET data were corrected for attenuation, scatter, randoms, decay, and dead time.

### Network architecture, data preprocessing, and training

To generate synthetic PET images, we applied an image-to-image translation network using a modified TensorFlow version of the pix2pixHD. We recently successfully employed and optimized this network architecture for denoising and enhancement of ultra-fast [F]FDG-PET images [22]. It is based on the original Pix2PixHD (https://tcwang0509.github.io/pix2pixHD/) which was proposed by Wang et al. and is an advanced form of the pix2pix architecture (https://phillipi.github.io/pix2pix/) incorporating two generators capturing both local and global image features [23, 24].

**Fig. 1:**
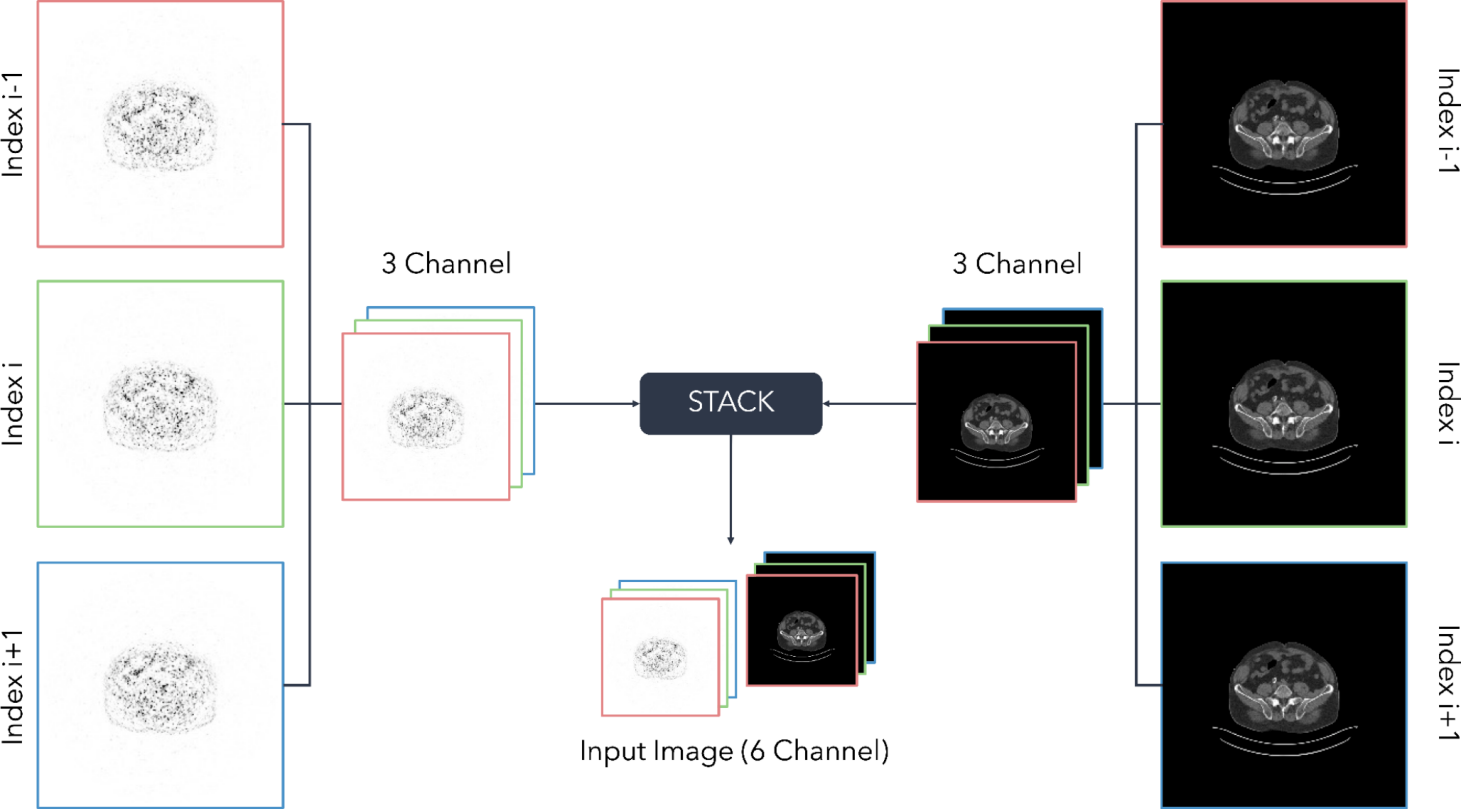
Visualization of the input slice generation process using the fast-scanned PET (left) and resampled CT scan (right). At the beginning, the previous and subsequent slices are combined with the slice based on the current index into a 3-channel image. In a final step, both modality-based 3 channel images were further combined into one 6-channel image representing the final input image

To generate 2.5-dimensional image input data sets, each axial slice of the ultra-fast PET and CT images was combined on the channel axis with its direct neighboring slices in cranial and caudal direction. Moreover, corresponding ultra-fast PET and CT images were combined into a final 6-channel input image for each slice index (schematic representation in Fig. 1) with a corresponding 1-channel target image from the full-time PET. PET voxel values were represented in units of body weight normalized standardized uptake values (SUV) and SUV values were normalized to an interval of (0, 1) using a constant maximum SUV of 50 as upper limit. CT images were adjusted to match the voxel size of the PET images and CT voxel values were normalized to an interval of (0, 1) based on Hounsfield units (HU) using − 1000 and 3000 as lower and upper limits, respectively. Resulting dimension of ultra-fast PET and CT input images was 224 × 224 pixels and for the training process the SUV/HU value range was projected from an interval of [0, 1] to [-1, 1].

**Fig. 2:**
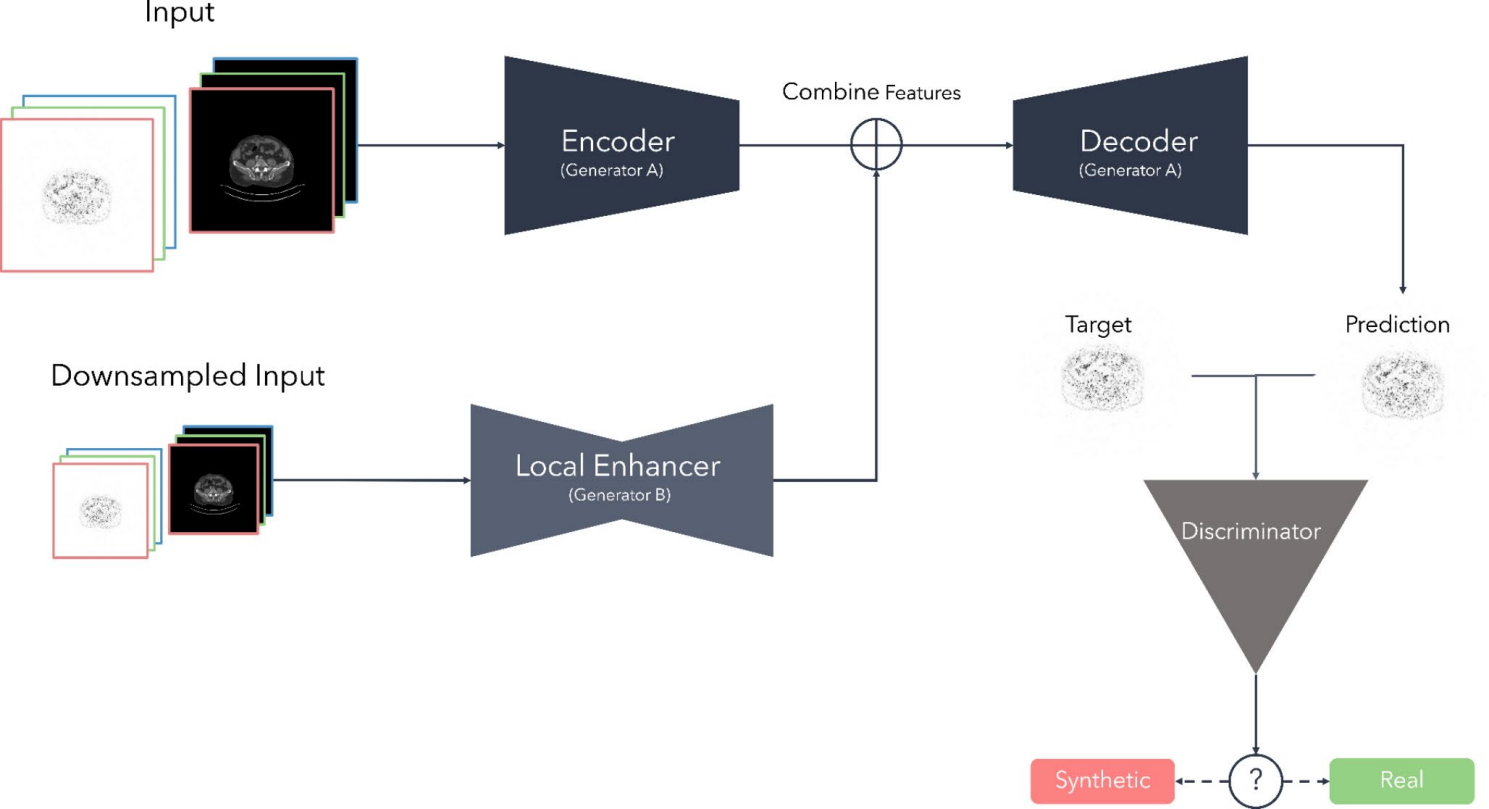
Design of the employed network architecture used for enhancing ultra-fast PSMA-PET acquisitions by incorporating a feature flow across two generators. The 6-channel input images are processed in two resolutions: they are sent in full resolution to the primary generator (Generator A) and, simultaneously, in a lower resolution to a local enhancer module (Generator B). Generator A captures the global structure of the input, while Generator B, the local enhancer, refines finer details by focusing on localized feature improvements in the reduced-resolution input. The feature maps generated by the local enhancer are then upscaled and merged with the feature maps from Generator A, allowing for an enhanced synthesis of high-resolution details in the final output. In the final stage, a discriminator evaluates the authenticity of the enhanced image, distinguishing it from the original

For the training process, the used GAN architecture is based on the enhancements and hyperparameter configurations described in Hosch et al. [22]. For this work, the model setup includes an average pooling operation within the first downsampling layers and a group convolution. Using group convolution, we aimed to achieve that PET and CT information and features are extracted separately for each modality in the first layer. During training, a batch size of four images was used and the output channel was configured to grayscale. All training runs were set to 100 epochs with an initial learning rate of 0.002 and a scheduled learning rate decay starting after 50 epochs.

Overall, five models were trained using a 5-fold cross-validation setup. In this approach, 80% (286 studies) of the data were used for training and 20% (71 studies) as a test cohort. During inference, each test sample was then used as input to all five models in an ensemble approach. In a final step, the five model predictions were averaged by a mean aggregation function over all voxels, resulting in a final prediction for each test sample.

### Image evaluation

The test cohort comprised 71 patient image datasets which were randomly selected. 11 patients (15.5%) of the test cohort underwent PSMA-PET imaging for primary staging, 38 (53.5%) in biochemical recurrence, 14 (19.7%) for restating of metastatic prostate cancer and 8 (11.3%) for evaluation of PSMA-based radionuclide therapy.

All ultra-fast, standard, and synthetic PET image data sets of the test cohort were manually evaluated in random order by a board-certified nuclear medicine physician with several years of experience in evaluation of PSMA-PET images. The reader was blinded to any clinical information. Each PET image data set was first evaluated in structured form using the PROMISE criteria scheme [25] and generating a structured miTNM reporting codeline. In brief, presence of tumor is evaluated in local (miT), pelvic lymph node (miN), and distant lymph node (miMa), bone (miMb), and distant organ (miMc) anatomical categories. Each category is divided into different subregions, i.e. sextant segmentation of the prostate gland, uptake in prostate bed after resection, as well as involvement of adjacent structures (left/right seminal vesicles or bladder) for miT, left/right internal iliac, left/right external iliac, left/right common iliac, left/right obturator, presacral, and other pelvic lymph nodes for miN, retroperitoneal, supradiaphragmatic, and other extrapelvic lymph nodes for miMa, unifocal, oligometastatic (*n* ≤ 3), or disseminated bone involvement for miMb, and lung, liver, or other distant organ involvement for miMc.

Moreover, the absolute number of lesions in each category (miT, miN, miMa, miMb, miMc) were reported and, for the lesion with the highest SUVmax value in each category, SUVmax (using standard PET images) and lesion size (using CT images) were measured. In the reporting process, the maximum number of counted lesions in each category was limited to 10 and bone lesions were regarded as not measurable.

### Comparison of lesion detectability and image quantification

For all detectability evaluations, the standard PET evaluation was used as gold standard and metrics were compared between ultra-fast and synthetic PET images. First, patient-based sensitivities, specificities, and accuracies for detection of lesions in miTNM regions were compared. Second, a detailed analysis of lesion detection in miTNM subregions was performed. In a patient-based analysis, the portion of patients with missed subregions and of patients with false-positive subregions was calculated. Moreover, in a subregion-based analysis, the mean number of missed subregions and of false-positive subregions per patient was calculated. Third, on a lesion-level, detection rates per miTNM region were compared.

In the analysis of quantitative performance, SUVmax values were compared both between ultra-fast and standard PET images and between synthetic and standard PET images. Moreover, differences in SUVmax values and lesion sizes were compared between miTNM regions that were correctly detected and miTNM regions that were not detected in the synthetic PET.

### Software and statistics

For all statistical computations, R statistical software in version 4.3.2 was used (R Foundation for Statistical Computing, Vienna, Austria, www.R-project.org*).* For comparison of sensitivities, specificities, and detection rates, a McNemar test was applied using the DTComPair package (https://cran.r-project.org/web/packages/DTComPair/); confidence intervals were calculated according to Altman et al. [26]. For comparison of lesion SUVmax values a paired t-test was applied. In all evaluations, *p*-values that were ≤ 0.05 were regarded as statistically significant. 

## Results

### Patient characteristics and descriptive analysis of the miTNM-Scores of the validation cohort

Patient characteristics of the validation cohort are shown by Table 1. Briefly, 23.9% of patients had local tumor, 31.0% local lymph node metastases, 22.5% distant lymph node metastases, 39.4% bone metastases and 5.6% visceral metastases.

**Table 1:** Patient characteristics

Characteristics	
Demographics	
Age (y) (mean ± SD)	69 ± 8
Weight (kg) (mean ± SD)	89 ± 17
Administered Activity (MBq) (mean ± SD)	344 ± 359
Imaging Delay (min) (mean ± SD)	55 ± 17
Indication for PET imaging	
Initial Staging	11 (15.5%)
Biochemical Recurrence	38 (53.5%)
Restaging in mPC	14 (19.7%)
Evaluation of PSMA-RLT	8 (11.3%)
miTNM	
T	
0	54 (76.1%)
2u	1 (1.4%)
2 m	2 (2.8%)
3a	1 (1.4%)
3b	1 (1.4%)
4	1 (1.4%)
r	11 (15.5%)
N	
0	49 (69.0%)
1	6 (8.5%)
2	16 (22.5%)
M	
0	36 (50.7%)
1a	16 (22.5%)
1b uni	10 (14.1%)
1b oligo	3 (4.2%)
1b diss	15 (21.1%)
1c	4 (5.6%)
PSA (ng/ml) (mean ± SD)	14.4 ± 37.1*

*PSA not available (within 3 months post/prior PET imaging) in 12 patients

### Evaluation of image quality

**Fig. 3:**
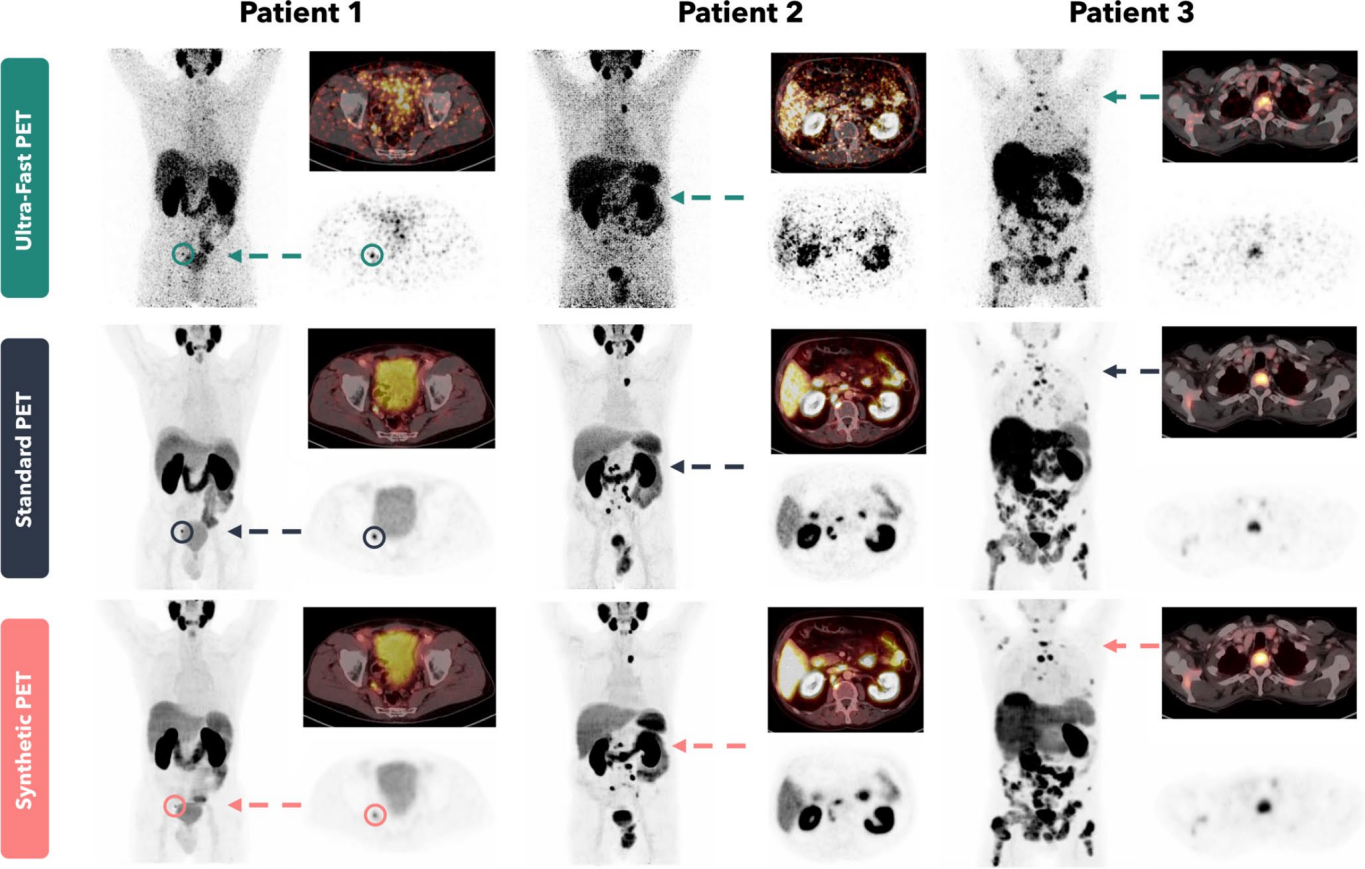
Image examples of three different patients. The images show anterior maximum intensity projections as well as axial PET and fused PET/CT images (SUV scale 0 to 10) of ultra-fast (top row), standard (middle row), and synthetic (bottom row) PET images. Arrows indicate the position of the axial slices. In patient 1 a lymph node metastasis (encircled for better visualization) was not detected in the ultra-fast PET but detected in the synthetic PET. In patient 2 reduction in image noise led to substantial improvement of image quality and lesion discernibility. In patient 3 the number of detected disseminated bone metastases was increased from the ultra-fast to the synthetic PET

Visual image quality was largely increased for the synthetic PET images compared with the ultra-fast PET images and resembled the image quality of the standard PET images. Improvements were, inter alia, reached by a reduction in image noise (see image examples in Fig. 3).

### Evaluation of miTNM codeline regions for ultra-fast PSMA-PET and synthetic PSMA-PET

The sensitivity, specificity, and accuracy of the ultra-fast PET and synthetic PET per miTNM region are shown by Table 2. For example, the sensitivity, specificity, and accuracy of the ultra-fast PET for local tumor were 58.8%, 100.0%, and 90.1%, respectively. This was improved by the synthetic PET with a sensitivity, specificity, and accuracy of 76.5%, 100.0%, and 94.4%, respectively.

**Table 2:** Patient-based sensitivity, specificity, and Accuracy for miTNM regions

	T	*N*	Ma	Mb	Mc
Sensitivity					
Ultra-fast PET (%)	58.8	59.1	68.8	75.0	50.0
Synthetic PET (%)	76.5	72.7	81.2	89.3	50.0
Difference	17.6	13.6	12.5	14.3	0.0
CI	-0.5–35.8	-0.7–28.0	-3.7–28.7	1.3–27.2	0.0
*P*-value (McNemar)	0.083	0.083	0.157	< 0.05	N/A
Specificity					
Ultra-fast PET (%)	100.0	100.0	100.0	100.0	50.0
Synthetic PET (%)	100.0	100.0	100.0	93.0	50.0
Difference	0	0	0	-7.0	0.0
CI	0	0	0	-14.6–0.6	0.0
*P*-value (McNemar)	N/A	N/A	N/A	0.083	N/A
Accuracy					
Ultra-fast PET (%)	90.1	87.3	68.8	90.1	97.2
Synthetic PET (%)	94.4	91.2	95.8	91.6	97.2

Overall, sensitivity was improved from ultra-fast PET to synthetic PET for local tumor (T), local lymph nodes (N), distant lymph nodes (M1a), and bone metastases (M1b) by a mean of 14.5%. For the M1b region, the sensitivity of synthetic PET was significantly higher (89.3 vs. 75.0, *p* < 0.05). However, for local tumor, local lymph nodes or distant lymph nodes statistical significance was not reached.

### Evaluation of miTNM codeline subregions for ultra-fast PSMA-PET and synthetic PSMA-PET

For all miTNM categories, the number of patients with missed subregions and the mean number of missed subregions per patient was improved for the synthetic PET (details are provided by Table S1 and Table 3).

**Table 3:** Patient-based analysis of miTNM sub-regions *

	Reference PET	Ultra-fast PET	Synthetic PET
T			
Patients with ≥ 1 positive subregion (n)	17		
Patients with ≥ 1 missed subregion (n)		8	5
Portion of patients with ≥ 1 missed subregion (reference: all patients / patients with ≥ 1 positive subregion)		11.6% / 47.1%	7.1% / 29.4%
Patients with ≥ 1 false-positive subregion (n)		0	0
Portion of patients with ≥ 1 false-positive subregion (reference: all patients / patients with ≥ 1 positive subregion)		0 / 0	0 / 0
N			
Patients with ≥ 1 positive subregion (n)	22		
Patients with ≥ 1 missed subregion (n)		17	12
Portion of patients with ≥ 1 missed subregion (reference: all patients / patients with ≥ 1 positive subregion)		23.9% / 77.7%	16.9% / 54.5%
Patients with ≥ 1 false-positive subregion (n)		1	1
Portion of patients with ≥ 1 false-positive subregion (reference: all patients / patients with ≥ 1 positive subregion)		1.4% / 4.5%	1.4% / 4.5%
M1a			
Patients with ≥ 1 positive subregion (n)	15		
Patients with ≥ 1 missed subregion (n)		5	5
Portion of patients with ≥ 1 missed subregion (reference: all patients / patients with ≥ 1 positive subregion)		7.0% / 33.3%	7.0% / 33.3%
Patients with ≥ 1 false-positive subregion (n)		0	1
Portion of patients with ≥ 1 false-positive subregion (reference: all patients / patients with ≥ 1 positive subregion)		0	1.4% / 6.7%
M1c			
Patients with ≥ 1 positive subregion (n)	5		
Patients with ≥ 1 missed subregion (n)		3	2
Portion of patients with ≥ 1 missed subregion (reference: all patients / patients with ≥ 1 positive subregion)		4.2% / 60.0%	2.8% / 40.0%
Patients with ≥ 1 false-positive subregion (n)		0	0
Portion of patients with ≥ 1 false-positive subregion (reference: all patients / patients with ≥ 1 positive subregion)		0	0

*for M1b, no subregions are defined

The highest number of missed subregions in mean was in the local lymph node category with 1.5 and 1.1 for ultra-fast and synthetic PET, respectively. In this category, ultra-fast PET missed a total of 10 subregions, whereas synthetic PET missed only a total of 7 regions (Supplemental Table S1). In patients with positive subregions, the mean number of missed lesions per patient was 0.59 (ultra-fast PET) and 0.41 (synthetic PET), respectively. Both ultra-fast PET and synthetic PET induced no false-positive T subregion.

### Evaluation of lesion detection rate per miTNM region for ultra-fast PSMA-PET and synthetic PSMA-PET

**Fig. 4:**
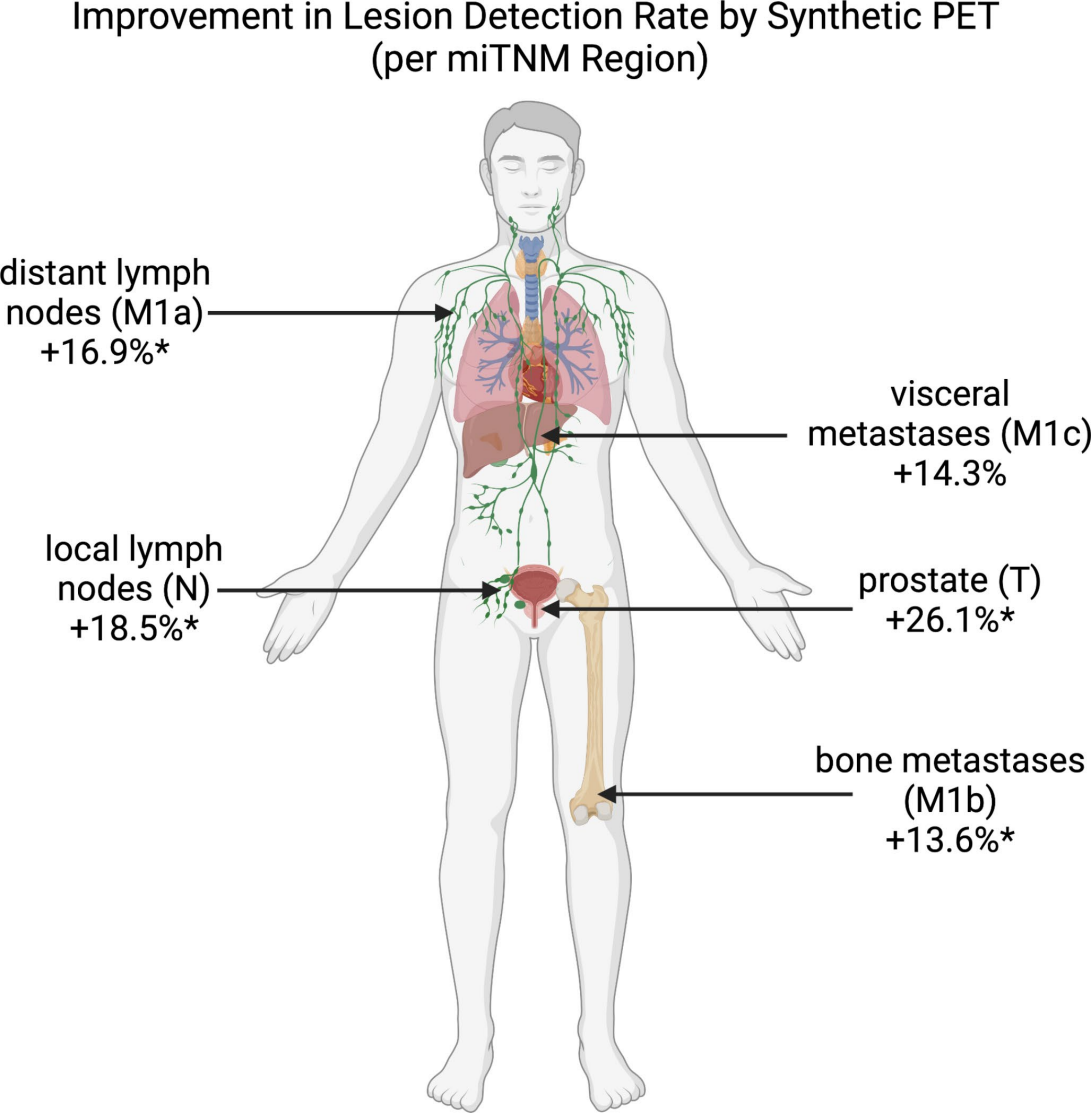
Visualization of lesion detection rate improvement by the neural network. The asterisks denote statistically significant improvements of lesion detection rate. The figure was created using BioRender.com (BioRender, San Francisco, USA, www.BioRender.com)

For all anatomical locations, the detection rate of the synthetic PET was significantly higher than the ultra-fast PET, except for M1c, which were only 7 lesions in total. In mean, the region-based detection rates were improved by 17.9%. Details are shown by Table 4. For example, the mean detection rate for the local tumor region was significantly higher for the synthetic PET compared with the ultra-fast PET (69.6 vs. 43.5, *p* < 0.05). For bone metastases, the detection rate was also significantly higher for the synthetic PET vs. ultra-fast PET (42.9 vs. 28.6, *p* < 0.05). An illustration of the improvements in detection rate is shown by Fig. 4.

**Table 4:** Lesion-based detection rates per miTNM region

	Detection rates (%) of				
	T	N	Ma	Mb	Mc
Ultra-fast PET	43.5	27.8	47.5	72.1	28.6
Synthetic PET	69.6	46.3	64.4	85.7	42.9
Difference	26.1	18.5	16.9	13.6	14.3
CI	8.1–44.0	11.2–25.8	10.2–23.7	8.1–19.1	-11.6–40.2
*P*-value (McNemar)	< 0.05	< 0.001	< 0.001	< 0.001	0.317

### Reproduction of SUV metrics comparing real, ultra-fast and synthetic PSMA-PET

Over all segmented tumor lesions, the SUVmax of the standard PET and the ultra-fast PET were significantly different (for example local tumor T1: 38.2 vs. 89.2, *p* < 0.001). This was improved by the synthetic PET with only the T category exhibiting a significant difference (38.2 vs. 24.7, *p* < 0.01), whereas the other categories showed no significant differences (for example, nodal: 29.1 vs. 26.1, *p* = 0.129). The results are shown separately for the miTNM regions in Table 5.

**Table 5:** Analysis of PET tumor quantification (SUVmax)

	Analyzed lesion pairs (*n*)	Full-dose PET	Ultra-fast PET	Synthetic PET	*p*-value Ultra-fast vs. Full-dose PET	*p*-value Synthetic PET vs. Full-dose PET
		(mean ± SD)				
T	11	38.2 ± 21.4	89.2 ± 46.7	24.7 ± 12.3	< 0.001	< 0.01
N	12	29.1 ± 14.8	51.1 ± 27.6	26.1 ± 11.9	< 0.01	0.129
M1a	12	30.6 ± 24.4	58.8 ± 46.1	19.8 ± 13.2	< 0.01	0.059
M1b	2	9.8 ± 2.2	20.2 ± 2.9	11.1 ± 1.2	< 0.001	0.937
M1c	21	24.1 ± 7.2	49.3 ± 21.8	24.2 ± 7.6	< 0.01	0.397

### Characterization of lesions missed in synthetic PET

In all evaluated miTNM regions, lesions missed in synthetic PET showed lower SUVmax values and lesion sizes (local tumor SUVmax: 8.1 vs. 31.8; diameter: 0.8 vs. 2.1 cm). See Table 6 for details.

**Table 6:** Characterisation of lesions missed in synthetic PET

	T	*N*	Ma	Mb	Mc
SUVmax*	(mean ± SD)				
Detected	31.8 ± 23.2	26.5 ± 14.7	29.3 ± 23.9	21.5 ± 8.9	9.8 ± 2.2
Not Detected	8.1 ± 2.8	9.8 ± 3.4	9.0 ± 1.6	6.5 ± 2.6	7.9 ± 0.2
Size (cm)**	(mean ± SD)				
Detected	2.1 ± 1.2	1.0 ± 0.7	0.9 ± 0.5	-	3.8 ± 0.7
Not Detected	0.8 ± 0.5	0.3 ± 0.1	0.4 ± 0.1	-	0.5 ± 0

* SUVmax values were measured in standard PET images

** Lesion sizes were measured in CT images, sizes of bone lesions (Mb) were regarded as not measurable to avoid selection bias, as these are often osteoblastic or sclerotic in patients with prostate cancer and, therefore, not measurable in accordance to RECIST criteria

## Discussion

In this study, the feasibility and effectiveness of ultra-fast [^68^Ga]Ga-PSMA-11 PET acquisitions with a digital PET/CT scanner and AI-based image post-processing was evaluated. The results suggest that this approach can produce high-quality PSMA-PET images at PET acquisition times comparable to a CT scan. The AI-based post-reconstruction significantly improved image quality and detection rates by a mean factor of 17.9% of tumor lesions. Yet, missed detection of lesions, especially in the relevant M1c category still limits extensive clinical application.

This is the, to the best of our knowledge, first description of AI-based post-processing for ultra-fast (i.e. table speed of 50 mm/s) PSMA-PET images and also for [^68^Ga]-based PET images. Several approaches were described for the enhancement of fast [^18^F]FDG-PET. However, these mostly targeted a moderate reduction in PET acquisition time by a factor of 4 to 10 (in contrast to a factor of approximately 40 which was used here). Moreover, one FDA-approved software product for enhancement of up to 4-fold reduced [^18^F]FDG-PET is already commercially available (https://subtlemedical.com/subtle-medical-receives-fda-510k-clearance-and-ce-mark-approval-for-subtlepet/). Here, a reduction in acquisition time was used that was higher by almost one magnitude than in most previous approaches.

We recently used a comparable setting for post-reconstruction of [^18^F]FDG-PET data. We could demonstrate the feasibility of generating high-quality PET images from ultra low-dose PET data with patient- and lesion-based detection rates of 89% and 78% for PERCIST-measurable lesions [22]. However, lesion detectability was limited for small-volume and low-uptake lesions (for example, indicated by patient- and lesion-based detection rates of 36% and 22% for non-PERCIST-measurable lesions). PSMA-PET is used in a far smaller number of clinical indications than FDG-PET, mostly comprising staging and re-staging of prostate cancer patients. This leads to a more limited ensemble of possible patterns in PSMA-PET images which are potentially easier to learn for a convolutional network. Moreover, clinically relevant findings can be summarized by the standardized miTNM report which facilitates the assessment of clinically relevant accuracy improvements [8].

Reproduction of miTNM reports, detection rates, and SUV metrics were significantly improved in the synthetic PSMA-PET compared to the ultra-fast PET. For example, detection rates were improved by 13.6–26.1% depending on the anatomical region (Fig. 4). This indicates the value of the AI-based approaches for substantial improvement of low dose PSMA-PET images. The improvement in detection rate for M1c lesions was not significant (Table 4) and patient-based sensitivity was not improved for M1c (Table 2). However, this might be an effect of low statistical power with only 7 lesions in the M1c category in 4 patients in the validation cohort. Future studies in larger cohorts may evaluate the potential of the AI-based post-reconstruction in this lesion group.

Overall, the sensitivity of the synthetic PET was yet limited for local, nodal, and distant lymph node miTNM findings with values of 77%, 73%, and 81%, respectively. These results are comparable to the previous examination we performed in [^18^F]FDG-PET imaging [22]. Whereas a clinical application of synthetic PSMA-PET in patients with biochemical PSA value recurrence, in which identification of single lesions is crucial, is not possible because of limited sensitivity (Table 2), a potential use case is frequent re-stating of patients with high PSMA-avid tumor burden undergoing PSMA radioligand therapy, in whom assessment of therapy response might be possible [27]. Typical examples of patients with high tumor burden in whom the AI-based approach led to a comparable assessment of tumor load to the standard PET images are shown in Fig. 3 (patients 2 and 3). In these scenarios the synthetic PET images could be used for monitoring of therapy response.

Detectability was particularly limited for lesions with low PSMA uptake (Table 6). This is, on the one hand, an expected finding, as these lesions are hardly to discriminate from enhanced image noise in ultra-low-dose PET images. This is a comparable setting to PET imaging using radionuclides with low positron branching ratios like ^124^I that produce noisy images and for which the minimum detectable activity negatively correlates with the lesion size [15]. On the other hand, patients undergoing PSMA radioligand therapy typically present themselves with high PSMA uptake. Thus, this does not generally impede a clinical application. Moreover, a crucial point is the substantial improvement in reproducibility of SUVmax values in the synthetic PET images (Table 5). Tumor uptake can be decisive to select patients for PSMA radioligand therapy and, therefore, plays a significant role in PSMA-PET imaging. Interestingly, the variations in SUVmax values were lower for the synthetic PET than for the ultra-fast PET and comparable to the full-dose PET (Table 5). A possible explanation for the large variation of SUVmax values in the ultra-fast PET may lie in the poor counting statistics at the ultra-short acquisition time.

The potential of the AI-based ultra-fast PET imaging approach may, in future studies, clinically be exploited by using longer acquisition times. These show lower image noise and higher lesion signal and might, therefore, lead to a lower rate of missed detections. For example, a 5-fold longer PET acquisition time (corresponding to a total acquisition time of 2–3 min) might significantly increase the diagnostic quality in synthetic PET images but still be highly beneficial for patient throughput and efficiency in clinical practice. For a PET scan duration of a few minutes, still not the acquisition time but other factors like patient turnover and positioning will be limiting for patient throughput. Other advantages of short-acquisition PET are increased patient comfort and reduction of motion artifacts [28].

An alternative to reducing PET scan times is decreasing the injected activity. As both measures correlate, in a first approximation, linearly [29], a post-reconstruction network trained on short-acquisition PET images can also be used for low-activity PET images. This could reduce radiation exposure for both medical staff and patients. An interesting future application of ultra-low dose PET might be PET-based screening. Moreover, reduced activities can increase cost-effectiveness and overcome shortcomings evoked by limited ^68^Ge/^68^Ga generator yield.

Future improvements of the post-reconstruction quality might be achieved by using optimized neural networks. Recently, cycleGANs were introduced to medical imaging [30]. These might improve a relevant limitation of the approach in this study: The pix2pixHD network is based on voxel-by-voxel matching of input and output images which might induce an error as the input standard and ultra-fast PET were separately acquired and not perfectly co-registered. However, the results were yet convincing taking into consideration the extremely low acquisition time. On the other hand, perfect co-registration is not required by cycleGANs making these promising candidates for future alternative neural network designs. Another network regime that should be investigated in future research are diffusion models for paired image-to-image translation tasks. Diffusion models have attracted considerable attention for their ability to generate high quality images through iterative refinement [31, 32]. They offer notable advantages in producing diverse outputs and handling complex generative tasks, making them well-suited to tasks with significant variability or inherent misalignment [31]. In contrast, GANs such as Pix2PixHD are specifically designed for structured, paired tasks and are more computational efficient (in terms of model parameters) in the described scenarios. Although diffusion models require significant computational resources and have been less explored for paired tasks, they show promise [33]. Their ability to produce fine-grained images suggests that, with further methodological advances, they could complement or replace existing GAN-based frameworks [34]. Further research into the adaptation of diffusion models for paired medical imaging translation tasks may open up new possibilities for achieving higher quality and more diverse results in this domain.

Another limitation is the single-center design of this prospective study using data from one digital PET system. Future multi-center studies are necessary to validate the results on external cohorts and using PET data acquired on scanners from different manufactures to pave the way for broader clinical applications. Moreover, the full-dose PET was used as reference standard since histopathological validation was beyond the scope of this study. PSMA-PET imaging is clinically well-established for staging of patients with prostate cancer.

## Conclusion

AI-based enhancement of ultra-fast PSMA-PET scans that were acquired approximately 40 times faster than standard PET is feasible and leads to significant improvements in image quality, lesion detectability, and image quantification. However, the sensitivity of the synthetic PET compared with the reference standard PSMA-PET is still limited. A possible clinical application could be disease monitoring in patients with high tumor load undergoing PSMA radioligand therapy. Not significantly improved detectability of distant metastases in synthetic PET compared with ultra-fast PET indicates missing training data for further optimization of the method.

## Electronic supplementary material

Below is the link to the electronic supplementary material.

Supplementary Material 1

## Data Availability

The datasets generated and/or analyzed during the current study are not publicly available due to privacy legislation but may be made available to qualified researchers on reasonable request from the corresponding author.
