# Supplementary material for: Staging of prostate Cancer with ultra-fast PSMA-PET scans enhanced by AI
Source: Eur J Nucl Med Mol Imaging. 2025 Jan 11;52(5):1658–70. doi: 10.1007/s00259-024-07060-7 (PMC11928425; doi:10.1007/s00259-024-07060-7)
Supplement: Supplementary file 1 — Supplementary Material 1 [file 259_2024_7060_MOESM1_ESM.pdf]

Supplemental Material to:

Staging of Prostate Cancer with Ultra-fast PSMA-PET scans Enhanced by AI

David Kersting*[,1], Katarzyna Borys*[,2,3], Alina Küper[1], Moon Kim[2], Johannes Haubold[2,3], Tsepo Goerttler[1], Lale Umutlu[3], Pedro Fragoso Costa[1], Jens Kleesiek[2], Christoph Rischpler[1,4], Felix Nensa[2,3], Ken Herrmann[1], Wolfgang P. Fendler[1], Manuel Weber[1], René Hosch*[,2,3], Robert Seifert*[,1,5]

* shared contribution

[1] Department of Nuclear Medicine and German Cancer Consortium (DKTK), University Hospital Essen, University of Duisburg-Essen, Essen, Germany

[2] Institute for Artificial Intelligence in Medicine, University Hospital Essen, Essen, Germany

[3] Institute of Interventional and Diagnostic Radiology and Neuroradiology, University Hospital Essen, Essen, Germany

[4] Department of Nuclear Medicine, Klinikum Stuttgart, Stuttgart, Germany

[5] Department of Nuclear Medicine, University Hospital Bern, University of Bern, Bern, Switzerland

Table S1: Detailed subregion-based Analysis (for M1b, no subregions are defined)

	Reference PET	Ultra-fast PET	Synthetic PET
**T**			
Positive subregions (n)	38		
Missed subregions (n)		10	7
Mean number of missed subregions per patient (reference: all patients / patients with ≥1 positive subregion)		0.14 (0.59)	0.09 (0.41)
False-positive subregions (n)		0	0
Mean number of false-positive subregions per patient (reference: all patients / patients with ≥1 positive subregion)		0 (0)	0 (0)
**N**			
Positive subregions (n)	59		
Missed subregions (n)		33	25
Mean number of missed subregions per patient (reference: all patients / patients with ≥1 positive subregion)		0.46 (1.5)	0.35 (1.1)
False-positive subregions (n)		1	1
Mean number of false-positive subregions per patient (reference: all patients / patients with ≥1 positive subregion)		0.01 (0.05)	0.01 (0.05)
**M1a**			
Positive subregions (n)	15		
Missed subregions (n)		5	5
Mean number of missed subregions per patient (reference: all patients / patients with ≥1 positive subregion)		0.07 (0.21)	0.07 (0.21)
False-positive subregions (n)		0	1
Mean number of false-positive subregions per patient (reference: all patients / patients with ≥1 positive subregion)		0	0.01 (0.04)
**M1c**			
Positive subregions (n)	5		
Missed subregions (n)		3	2
Mean number of missed subregions per patient (reference: all patients / patients with ≥1 positive subregion)		0.04 (0.18)	0.03 (0.12)
False-positive subregions (n)		0	0
Mean number of false-positive subregions per patient (reference: all patients / patients with ≥1 positive subregion)		0	0
